# HIV and immunotherapy: correspondence

**DOI:** 10.1097/JS9.0000000000001073

**Published:** 2024-01-11

**Authors:** Hinpetch Daungsupawong, Viroj Wiwanitkit

**Affiliations:** a Private Academic Consultant, Phonhong, Lao People’s Democratic Republic; b Department of Research Analytics, Saveetha Dental College and Hospitals, Saveetha Institute of Medical and Technical Sciences, Saveetha University, Chennai, Tamil Nadu, India

*Dear Editor*,

We would like to share ideas on the publication ‘HIV and immunotherapy: will CAR-T cell therapy cure HIV?^1^.’ This intriguing question was posed and debated by Veeraraghavan *et al*. Hundreds of thousands of people worldwide are dying from AIDS-related illnesses, and millions more are living with HIV. As a result, the burden of AIDS and its repercussions is growing. New and more potent therapeutic strategies, such as chimeric antigen receptor T-cell (CAR-T) cell therapy, are being developed to counter this. CAR-T cell therapy is currently being researched as a possible HIV treatment because it has demonstrated encouraging results in treating several forms of cancer. CAR-T cells can proliferate and engraft, in contrast to traditional therapies, offering a long-lasting therapeutic impact and the possibility of totally eliminating HIV-infected cells. Though this method has demonstrated promising results in the laboratory, clinical trials have not yet validated its therapeutic advantages.

The goal of CAR-T cell treatment is to alter a patient’s own T cells so that they express chimeric antigen receptors, which are particularly designed to identify and attack HIV-positive cells. After being reinfused into the patient, these altered T cells are able to identify and eliminate HIV-positive cells. Although CAR-T cell therapy has demonstrated remarkable efficacy in treating specific tumors, it encounters considerable obstacles when it comes to treating HIV. One difficulty is that HIV is a persistent and dynamic virus that evades identification by CAR-T cells. Furthermore, HIV reservoirs, which are concealed areas of viral replication that are difficult for the immune system to access, may not be efficiently targeted by CAR-T cell treatment. It is likely that CAR-T cell treatment will need to be used in conjunction with other antiretroviral medications or immune-based therapies in order to effectively treat HIV. Combination therapies may result in long-term viral suppression and control, enabling those living with HIV to lead healthy lives without the requirement for ongoing antiretroviral medication.

## Ethical approval

Not applicable.

## Consent

Not applicable.

## Sources of funding

None.

## Author contribution

H.D.: 50% ideas, writing, analyzing, and approval; V.W.: 50% ideas, supervision, and approval.

## Conflicts of interest disclosure

Authors declare no conflicts of interest.

## Research registration unique identifying number (UIN)

Not applicable.

## Guarantor

Professor Viroj Wiwanitkit.

## Data availability statement

There are no new data generated.

## Provenance and peer review

Not applicable.
